# Supplementary material for: Complex‐centric proteome profiling by SEC‐SWATH‐MS
Source: Mol Syst Biol. 2019 Jan 14;15(1):e8438. doi: 10.15252/msb.20188438 (PMC6346213; doi:10.15252/msb.20188438)
Supplement: Supplementary file 6 — Dataset EV5 [file MSB-15-e8438-s006.zip › feature_plots_corum/5378.pdf]

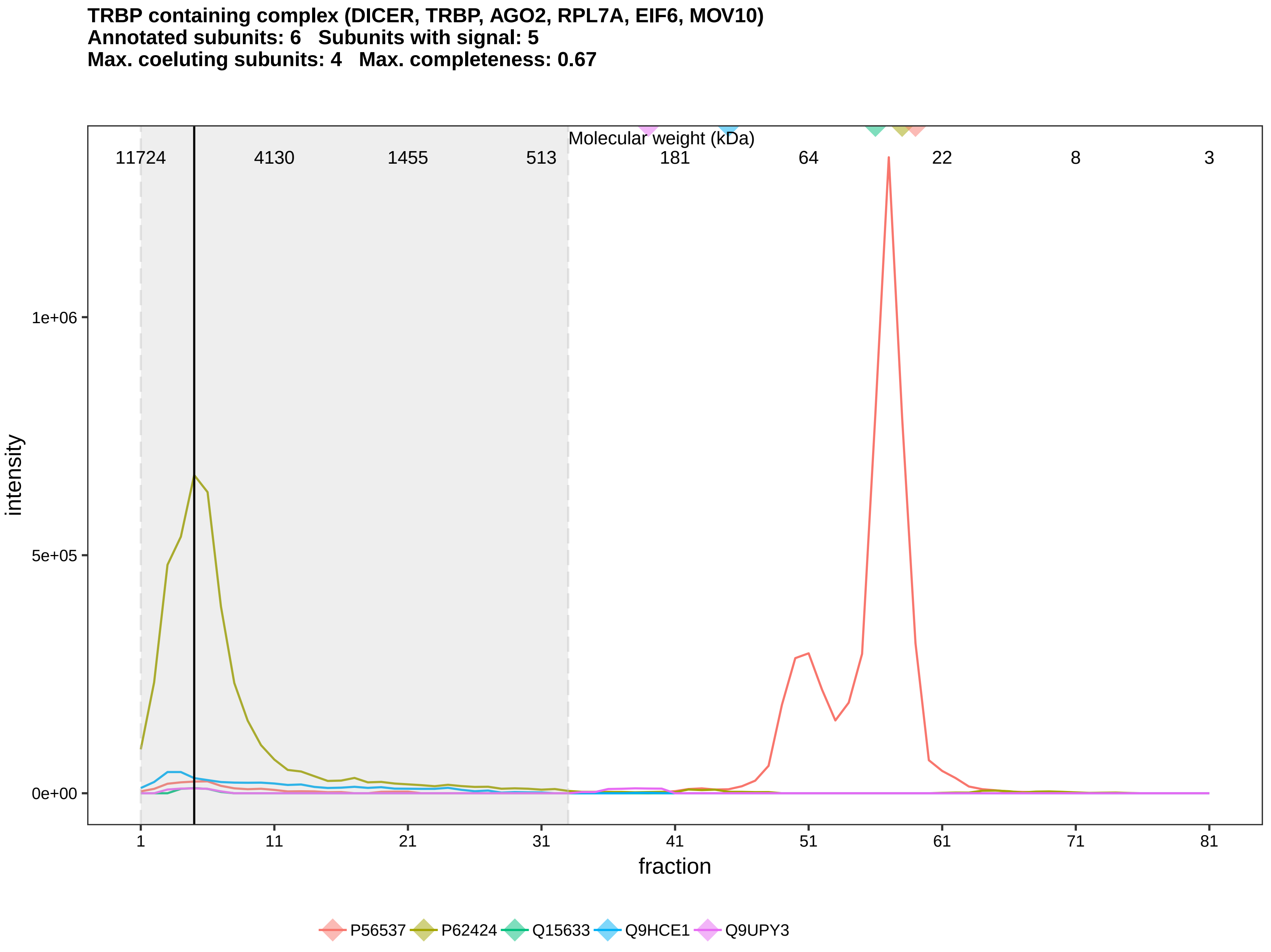

TRBP containing complex (DICER, TRBP, AGO2, RPL7A, EIF6, MOV10)
Annotated subunits: 6 Subunits with signal: 5
Max. coeluting subunits: 4 Max. completeness: 0.67
Molecular weight (kDa)
11724
4130
1455
513
181
64
22
8
3
intensity
1e+06
5e+05
0e+00
fraction
1
11
21
31
41
51
61
71
81
P56537
P62424
Q15633
Q9HCE1
Q9UPY3
